# GABA_B_ receptor regulates proliferation in the high-grade chondrosarcoma cell line OUMS-27 via apoptotic pathways

**DOI:** 10.1186/s12885-018-4149-4

**Published:** 2018-03-07

**Authors:** Kiyoto Kanbara, Yoshinori Otsuki, Masahito Watanabe, Syunichi Yokoe, Yoshiaki Mori, Michio Asahi, Masashi Neo

**Affiliations:** 1 Department of Orthopedics, Osaka Medical College Takatsuki, Daigaku-machi 2-7, Takatsuki, Osaka, 569-8686 Japan; 2 President of Osaka Medical College, Daigaku-machi, Takatsuki, Osaka, Japan; 3 Department of Rehabilitation Sciences, Faculty of Allied Health Sciences, Kansai University of Welfare Sciences, Asahigaoka, Kashihara, Osaka, Japan; 4 Department of Pharmacology, Faculty of Medicine, Osaka Medical College, Daigaku-machi, Takatsuki, Osaka, Japan

**Keywords:** Chondrosarcoma, GABA_B_ receptor, Akt signaling, Whole cell patch clamp, Ca^2+^ channel, Apoptosis, MAPK pathway, Cell cycle arrest, OUMS-27 cells

## Abstract

**Background:**

High-grade chondrosarcoma, which has a high incidence of local recurrence and pulmonary metastasis despite surgical resection, is associated with poor prognosis. Therefore, new and effective adjuvant therapies are urgently required for this disease. Gamma-aminobutyric acid (GABA), which acts as a neurotrophic factor during nervous system development, is related to the proliferation and migration of certain cancer cells. The GABAergic system, which is composed of GABA, the GABA-synthesizing enzyme glutamic acid decarboxylase (GAD), and GABA receptors, has an important function in nerve growth and development of neural crest. Therefore, the GABAergic system may play important functional roles in the proliferation of chondrosarcoma cells, which are derived from neural crest cells. We examined the anti-tumor effects of the GABAergic system on a chondrosarcoma cell line.

**Methods:**

We evaluated the underlying mechanisms of the anti-tumor effects of the GABAergic system, such as the involvement of different signaling pathways, apoptosis, and cell cycle arrest, in the high-grade chondrosarcoma cell line OUMS-27. In addition, we performed whole-cell patch-clamp recordings for Ca^2+^ currents and evaluated the changes in intracellular Ca^2+^ concentration via Ca^2+^ channels, which are related to the GABA_B_ receptor in high-grade chondrosarcoma cells.

**Results:**

The GABA_B_ receptor antagonist CGP had anti-tumor effects on high-grade chondrosarcoma cells in a dose-dependent manner. The activities of caspase 3 and caspase 9 were significantly elevated in CGP-treated cells compared to in untreated cells. The activity of caspase 8 did not differ significantly between untreated cells and CGP-treated cells. However, caspase 8 tended to be up-regulated in CGP-treated cells. The GABA_B_ receptor antagonist exhibited anti-tumor effects at the G1/S cell cycle checkpoint and induced apoptosis via dual inhibition of the PI3/Akt/mTOR and MAPK signaling pathways. Furthermore, the changes in intracellular Ca^2+^ via GABA_B_ receptor-related Ca^2+^ channels inhibited the proliferation of high-grade chondrosarcoma cells by inducing and modulating apoptotic pathways.

**Conclusions:**

The GABA_B_ receptor antagonist may improve the prognosis of high-grade chondrosarcoma by exerting anti-tumor effects via different signaling pathways, apoptosis, cell cycle arrest, and Ca^2+^ channels in high-grade chondrosarcoma cells.

## Background

Chondrosarcoma can be defined as a malignant chondrogenic tumor characterized by the formation of cartilaginous neoplastic tissue and accounts for 20% of all malignant bone tumors [1]. The prognoses are strongly correlated with histologic grading. Generally, chondrosarcoma is considered as relatively resistant to conventional chemotherapy and radiation therapy, and adequate surgical resection is effective for low-grade chondrosarcoma. In contrast, high-grade chondrosarcoma is associated with poor prognosis because it has a high incidence of local recurrence and metastasis to the lung, despite surgical resection. Recently, gene therapy with a Bcl inhibitor [2] and tyrosine kinase inhibitor [3] and knockdown of genes encoding metalloproteinases or regulators of histone acetylation/deacetylation [4] were performed in chondrosarcoma. Further, prognostic improvement in high-grade chondrosarcoma is expected when using a combination of these anti-tumor immunotherapeutic approaches and surgical treatment. GABA, the principal inhibitory neurotransmitter in the central nervous system, acts as a neurotrophic factor during embryonic development of the nervous system [5–7]. For example, GABA plays important roles in proliferation, migration, and differentiation during nervous system development [8–11]. Previous studies showed that embryonic neural cells and neural crest cells produce GABA and the GABA-synthesizing enzyme GAD during embryonic development [12–14]. GABA and GABA receptors have also been detected in numerous peripheral non-neuronal tissues, including cartilaginous tissue. Rat growth plate chondrocytes express GABA receptors, and activation of GABA receptors promotes the proliferation of mouse chondrogenic ATDC5 cells [10, 15]. The physiological effects of GABA are exerted via GABA receptors [7]. There are three types of GABA receptors: ionotropic GABA_A_, and GABA_C_ receptors and metabotropic G protein-coupled GABA_B_ receptors [16, 17].

Chondrosarcoma has embryonic origin from neural crest cells outside the mesoderm [18, 19]. Expression of the GABAergic system, composed of GABA, GAD, and GABA receptors, was observed in certain cancers such as human colon cancer, breast cancer, gastric cancer, and prostate cancer, among others [20]. These reports indicated a relationship between the GABAergic system and oncogenesis in cancer cell proliferation. GABA is an important nerve growth factor required for the development of neural crest cells and may also affect chondrosarcoma proliferation. Therefore, we examined the anti-tumor effects of the GABAergic system in a chondrosarcoma cell line. Further, we evaluated the involvement of various mechanisms of anti-proliferation, such as signaling pathways, apoptosis, and cell cycle arrest, in a chondrosarcoma cell line. Elucidation of the relationship between the GABAergic system and high-grade chondrosarcomas can aid in the development of new therapies for high-grade chondrosarcoma.

## Methods

### Cell culture

The human high-grade chondrosarcoma cell line OUMS-27 [21], characterized by short tandem repeat analysis (access code; CVCL_3090), was obtained from Okayama University.

Cells were grown in supplemented Dulbecco’s modified Eagle medium with 10% (*v*/*v*) heat inactivated fetal bovine serum and 1% antibiotic-antimycotic (100×, Thermo Fisher Scientific, Waltham, MA, USA) under an atmosphere of 95% air and 5% CO_2_ at 37 °C. Cells were confirmed to be free of mycoplasma infection using an e-Myco Mycoplasma PCR Detection Kit (iNtRON Biotechnology, Gyeonggi-do, Korea).

### Immunohistochemical and fluorescence analyses

OUMS-27 cells were harvested by low speed centrifugation at 800 rpm and washed twice in phosphate-buffered saline (PBS) without trypsin. The harvested cells were fixed with 50 mL 4% (*w*/*v*) paraformaldehyde and 0.05% glutaraldehyde in 0.1 M phosphate buffer (PB, pH 7.4). Following brief rinsing with PBS, the cells were blocked with 3% bovine serum albumin/PBS. Immunohistochemistry was performed for the GABA_A_ receptor and its subunits, GAD, and GABA using a goat polyclonal antibody directed against the GABA_A_ receptor subunits α2, α3, β1, and γ3 and GABA_B_ receptor R1 subunit (diluted 250×; Santa Cruz Biotechnology, Inc., Dallas, TX, USA), rabbit polyclonal antibodies directed against GAD65 (diluted 1000×; Chemicon International, Temecula, CA, USA) and GABA (diluted 250×; Chemicon International), mouse monoclonal antibody directed against GAD67 (diluted 1000×; Chemicon International), and guinea pig polyclonal antibody directed against the GABA_B_ receptor R2 subunit (diluted 1500×; Chemicon International). The specificity of these antibodies has been reported previously [22–24]. Briefly, sections pre-washed with PBS were incubated with normal donkey or goat serum (diluted 50×) for 30 min at room temperature (RT), followed by overnight incubation with each primary antibody at 4 °C. The sections were rinsed with PBS and incubated with Alexa Fluor™ 488 donkey anti-goat IgG secondary antibody (diluted 300×; Molecular Probes, Eugene, OR, USA) for the GABA_A_ receptor subunits and GABA_B_ receptor R1 subunit, Alexa Fluor™ 488 goat anti-rabbit IgG (diluted 300×; Molecular Probes) for GABA and GAD65, Alexa Fluor™ 488 goat anti-mouse IgG (diluted 300×; Molecular Probes) for GAD67, and Alexa Fluor^R^ 546 goat anti-guinea pig IgG (diluted 300×; Molecular Probes) for the GABA_B_ receptor R2 subunit for 60 min at RT in the dark. Subsequently, the sections were rinsed with PBS. The sections, except those used for double-staining, were treated with 100 μg/mL RNase A in PBS for 1 h at 37°C and counterstained with 10 μg/mL of propidium iodide (PI, Molecular Probes) diluted in phosphoric and citric acid buffer for 3 min at RT. After several rinses with PBS, immunoreactivity was examined using a confocal laser microscope (LSM510, Zeiss Co., Ltd., Oberkochen, Germany) equipped with a 488-nm argon laser. Sections incubated with non-immune sera from the same species as the primary antibody served as negative controls.

### RNA isolation and reverse transcription-polymerase chain reaction (RT-PCR) of chondrosarcoma cells

OUMS-27 cells were harvested by low speed centrifugation at 800 rpm and washed three times in PBS without enzyme. Total RNA was extracted from the harvested cells using the RNeasy mini kit (Qiagen GmbH, Hilden, Germany). cDNA was synthesized using Omniscript reverse transcriptase (Qiagen GmbH) according to the manufacturer’s instructions. The reverse transcription reaction mixture contained 1 μM oligo-d (T)12–18 primer, 10 U RNase inhibitor, 0.5 mM of each dNTP, and 4 U Omniscript reverse transcriptase. PCR was performed in a GeneAmp PCR System 9700 thermal cycler (Applied Biosystems, Foster, CA, USA). The PCR reaction mixture (25 μL) contained 1× GoTaq Green Master Mix reaction buffer (pH 8.5), 400 μM dNTPs, 3 mM magnesium chloride_,_ (Promega, Madison, WI, USA), 2 μL cDNA solution, and 0.2 μM of each primer. The primer sequences used for GAD65, GAD67, the GABA_A_ α1–6, β1–3, γ1–3, δ, π, θ, and ε subunits, and GABA_B_ R1a, R1a/b, and R2 are shown in Table 1. The PCR products were separated on 1.5% agarose gels, followed by staining with 0.1% ethidium bromide solution for 10 min and illuminated using an ultraviolet transilluminator.

**Table 1:** Primers used for RT^_^PCR of human GABA receptor subunits and GADs

Target (accession no.)		Primer sequence (5^,_^3’)	Position	Length (bp)
GABA_A_α1 (NM_000806)	F	GGAATTGT C CAGT CAAGTACAGG	1150–1172	532
	R	TTGTTTCGGGCTTGACCTC	1663–1681	
GABA_A_α2 (NM_000807>	F	GCTTATGCAGTGGCTGTTGC	1411–1430	257
	R	GGACTGAC C C CTAATACAGGTTC	1645–1667	
GABA_A_α3 (NM_000808)	F	C CAC CT AT C C CAT CAAC CTG	1442-1461	261
	R	TGCCCTTGATAGCTGACTCC	1683–1702	
GABA_A_α4 (NM_000809)	F	AGACATCAAA GCCCCCTCAG	2044–2063	307
	R	GGAGAAGCAGATGGAAGTGC	2331–2350	
GABA_A_α5 (NM_000810)	F	CGCTTTTACAACTGGGAAGATG	1657–1678	277
	R	GAGTGTGGCCGGTTATTTTG	1914-1933	
GABA_A_α6 (NM_000811)	F	CCCACCCACA GTGACAATAT C	1367–1387	331
	R	TTCAACACTGCTACTGACTTCC	1676–1697	
GABA_A_β1 (NM_000812)	F	AATCCGGAATGAGACGAGTG	1493–1512	326
	R	GGAAC CATTAGAACAGAC CT CAG	1796–1818	
GABA_A_β2 (A28108)	F	TGCCAACAATGAGAAGATGC	1259–1278	401
	R	AGT GGGAGGC CAT GTTTTAG	1640–1659	
GABA_A_β3 (NM_000814)	F	GACCGTTCAAAGAGCGAAAG	1168-1187	233
	R	CGTAGATGGGTCTTCTTGTGC	1380-1400	
GABA_A_γ1 (NM_173536)	F	TAAAGCCTCG ATGACTCCTG	1274–1293	311
	R	GACTTCTTTTGATTTTTGCTTATGG	1560-1583	
GABA_A_γ2 (NM_000816)	F	TTTGTCAGCAACCGGAAAC	1433–1451	355
	R	C CATATCAGTAAAACCCATAC CTC	1764-1787	
GABA_A_γ3 (NM_033223)	F	C CAAC CAC CAC GAAGAAGAC	1305–1324	397
	R	GCTCTTCACTCTTCACTCTGAGC	1679–1701	
GABA_A_δ (NM_000815)	F	ATTT CAACGC CGACTAC AGG	1090–1109	300
	R	GGGCGTAAATGTCAATGGTG	1370–1389	
GABA_A_ε (NM_00496l)	F	GACAAAAG C C CAT G CTTCTC	1144-1163	255
	R	AAACGCTTGCACCACTCAC	1380–1398	
GABA_A_π (U95367)	F	GTTTGAGCTT CGGAGGAATG	861–880	201
	R	AAGCAGTTGGTGTTGGGAAG	1042–1061	
GABA_A_θ(BD106470)	F	CCTCAGCCCACTCACTTCTC	1278-1297	297
	R	GCTTCTTGCACACCCTTCTC	1555-1574	
GABA_B_R1a (AJ012185)	F	CAACGC CACCTCAGAAG	69–85	234
	R	GAGCAGATTCGGACACAG	285-302	
GABA_B_R1a/b (AJ012186)	F	CTGAATCCTGCCAATACCC	937–955	256
	R	AGTTCATTGCCCGGTAGA	1175-1192	
GABA_B_R2 (AJ012188)	F	GACCATCTCAGGAAAGACTC	984–1003	235
	R	GGTCTCGTTCATGGCATT	1201-1218	
GAD65 (NM_000818)	F	ATGC CT C CTACCT CTTT CAG	1406–1425	218
	R	CACCATCTCATATCCTTCTC	1604-1623	
GAD67 (NM_000817)	F	ACTGGCTGAATACCTCTATG	2005–2024	318
	R	CCCAGT CTTT CTATCTCCTC	2303-2322	
		F: forward; R^:^ reverse		

### Cell viability assays

OUMS-27 cells were plated at a concentration of 1 × 10^4^ cells/well on 96-well plates one day before each drug treatment. They were subsequently incubated for 48 h in culture medium containing one of the following GABA receptor ligands: 100 μM GABA and GABA_A_ receptor agonist, 50 μM Muscimol(MUS) GABA_B_ receptor agonists, 100 μM R-(+)-Baclofen(BFN)or 10 μM SKF 97541 [3-aminopropyl (methyl) phosphine acid] (SKF), 100 μM GABA+ GABA_A_ receptor antagonist, 100 μM (−)-bicuculline methochloride(BMC)or GABA_B_ receptor antagonist, 1 μM CGP54626(CGP). After seeding the cells, 5-bromo-2′- deoxyuridine (BrdU) was added to a final concentration of 1 μM in each sample and incubated for 2 h. Thereafter, DNA synthesis was assayed by cell proliferation enzyme-linked immunosorbent assay (ELISA) and BrdU (Roche Molecular Biochemicals, Basel, Switzerland) was estimated by colorimetric detection according to the manufacturer’s instructions. Colorimetric analysis was performed using an ELISA plate reader (VersaMax, Molecular Devices, Sunnyvale, CA, USA).

### Flow cytometric analysis of apoptosis

First, 1 × 10^6^ OUMS-27 cells, both adherent and suspended, harvested after 24 h treatment with various concentrations of CGP or dimethyl sulfoxide (DMSO) (10, 100, 250, 500 μM CGP), were fixed in 1% (*w*/*v*) paraformaldehyde in PBS (pH 7.4). The APO-DIRECT™ apoptosis detection kit (BD Biosciences, San Jose, CA, USA) was used according to the manufacturer’s protocol. The APO-DIRECT™ assay is a single-step method for labeling DNA breaks with FITC-dUTP. Cells isolated from each step were incubated at − 20 °C in 70% (*v*/*v*) ethanol for 30 min. Apoptotic cells were analyzed by FACScan flow cytometry and BD Diva Software Version 4.1 (BD Biosciences).

### Caspase activity

OUMS-27 cells were plated at concentration of 1 × 10^4^ cells/well on a 96-well plate one day before CGP treatment. Cell viability was measured after treatment with 10 μM CGP or DMSO for 24 h using the Cell Titer-Blue cell viability assay (Promega). The activities of caspase-3, caspase-8, and caspase-9 were measured using the Caspase-Glo™ Assay luminescence kit (Promega). The fluorescent intensities for the cell viability assay and luminescent intensities for caspase activity were measured using the GloMax-Multi detection system (Promega). The activity of each caspase was adjusted to account for the corresponding cell viability data as described previously [25].

### Flow cytometric analysis of cell cycle

OUMS-27 cells were grown in each well with various concentrations of CGP or DMSO for 24 h. Harvested cells were fixed in cold 70% (*v*/*v*) ethanol. CycleTEST™ PLUS DNA reagent kit (BD Biosciences) was used according to the manufacturer’s protocol. The cell cycle distribution in all sample cells was measured by flow cytometry (EPICS Elite ESP; Beckman Coulter Co., Brea, CA, USA) and the percentage of cells in each phase of the cell cycle was analyzed using a Multi Cycle for Windows Version 3.0 (PHOENIX, San Diego, CA, USA).

### Western blot analysis

After treatment with 10 μM CGP or control for 24 h, OUMS-27 cells were harvested and proteins were extracted. The cells were homogenized in 1 mM Tris-HCl (pH 7.5) containing 150 mM sodium chloride, 5 mM EDTA, 1% (*w*/*v*) sodium dodecyl sulfate (SDS), 1 mM phenylmethane sulfonyl fluoride, 1% (*w/v*) sodium deoxycholate, and 0.5% (*w/v*) protease inhibitor cocktail (Sigma Aldrich, St. Louis, MO, USA). After centrifugation, the protein concentration in the cell supernatants was determined using the Qubit^a^ protein assay kit (Molecular Probe) and a Qubit^a^.2.0 fluorometer. Aliquots containing 2.5 or 5 μg protein were then boiled in loading buffer containing 50 mM Tris (pH 6.8), 6% 2-mercaptoethanol, 2% SDS, 10% glycerol, and 0.004% bromophenol blue. Each aliquot was loaded onto an 8% polyacrylamide gel. After electrophoresis, the gels were transferred to polyvinylidene fluoride membranes (Bio-Rad Laboratories, Hercules, CA, USA). The membranes were incubated with 1% bovine serum albumin in TBS containing 0.1% Tween-20 overnight to block non-specific binding, followed by incubation with GAD65, GAD67, total Akt, phospho-Akt-Ser473, phospho-Akt-Thr308, ERK 1/2, phospho-ERK1/2, JNK, phospho-JNK, p38 (diluted 1000×, Cell Signaling Technology, Danvers, MA, USA), phospho-p38 (diluted 2500×, BD Biosciences) or anti-GAPDH antibodies (diluted 3200×, MAb 6C5, HyTest, Turku, Finland). After rinsing the membranes, horseradish peroxidase (HRP)-linked anti-rabbit IgG and HRP-linked anti-mouse IgG (Cell Signaling) secondary antibodies were applied and the chemiluminescent reaction was performed using ECL Plus western blotting detection reagents (GE Healthcare, Little Chalfont, UK). Protein expression was detected using the LAS-3000 Lumio image analyzer (Fuji Photo Film, Tokyo, Japan). Signal intensities were further analyzed using Multi Gauge software (version 3.0; Fuji Photo Film).

### Comprehensive cell cycle mRNA expression analysis by real-time PCR

OUMS-27 cells were treated with 10 μM CGP or DMSO for 24 h. Total RNA was extracted from each cell using the RNeasy mini kit (QIAGEN, Hilden, Germany), and cDNA was synthesized using QuantiTect reverse transcription kit (QIAGEN). Primers in the Human PrimerArray Cell Cycle series (Takara Bio, Inc., Shiga, Japan) were used to amplify loci involved in cell cycle regulation. Real-time PCR was performed with the Thermal Cycler Dice Real Time system (Takara Bio, Inc.) according to the manufacturer’s instructions. Data were normalized to the expression of glyceraldehyde-3- phosphate dehydrogenase and expressed as the mean ± standard deviation (SD).

### Ca^2+^ channel current recording

Whole-cell patch-clamp recordings for Ca^2+^ currents in OUMS-27 cells treated with 100 μM GABA and 1 μM CGP were conducted as previously described [26–28]. Patch pipettes were pulled from borosilicate glass capillary tubes (GC120F15, Clark Electromedical Instruments, Pangbourne, UK) using a vertical electrode puller (PP-83, Narishige Scientific Instrument Laboratories, Tokyo, Japan), which exhibited resistance between 4 and 6 MΩ. The filling solution of the patch-pipettes contained 80 mM cesium chloride, 65 mM cesium-methane sulfate, 2 mM EGTA, 10 mM HEPES, 4 mM ATP-Mg, and 0.2 mM GTP-Tris (pH 7.3). The bath solution for recording consisted of 135 mM tetraethylammonium chloride, 10 mM barium chloride, 10 mM HEPES, 10 mM D-glucose, and 20 mM sucrose (pH 7.3 adjusted with 1 N hydrochloric acid). Ca^2+^ channel currents were routinely evoked with a 100 ms voltage step from − 60 to 0 mV in increments of 10 mV. GABA-induced Ca^2+^ channel currents were measured using a patch-clamp amplifier (EPC-9, HEKA Elektronik, Lambrecht, Germany) which was controlled by a Macintosh computer (Power Macintosh G3, Apple Computer, Cupertino, CA, USA) and control software (Pulse + PulseFit, HEKA Elektronik, Lambrecht/Pfatz, Germany).

### Measurement of Ca^2+^ concentration in chondrosarcoma cells

The intracellular Ca^2+^ concentration, [Ca^2+^]_I,_ in drug-treated OUMS-27 cells, was measured by Fura-2 AM, a calcium-sensitive dye (Dojindo, Kumamoto, Japan). Briefly, OUMS-27 cells were loaded with 5 μM Fura-2 AM in loading buffer containing 0.01% pluronic F-127 (Dojindo) for 30 min at 37 °C. After washing Fura-2 AM out of the loading buffer, the relative transient calcium concentration (OD_340nm_/OD_380nm_ excitation ratio) was recorded before and after the addition of 10 mM GABA (Sigma-Aldrich) in a perfusion chamber using the AQUACOSMOS/RATIO, C7773 (Hamamatsu photonics, Hamamatsu, Japan). Recording was continued after washing the 10 mM GABA out of the buffer. [Ca^2+^]_I_ after pretreatment with 1 μM CGP and application of 10 mM GABA was recorded using a similar method.

### Statistical analysis

The results are shown as the mean ± standard deviation (SD) and *P* values less than 0.05^*^, 0.01**, or 0.001*** were considered statistically significant using Student’s *t*-tests. Each experiment was performed at least three times under identical conditions.

## Results

### Expression of the GABAergic system in high-grade chondrosarcoma cells

**Fig. 1:**
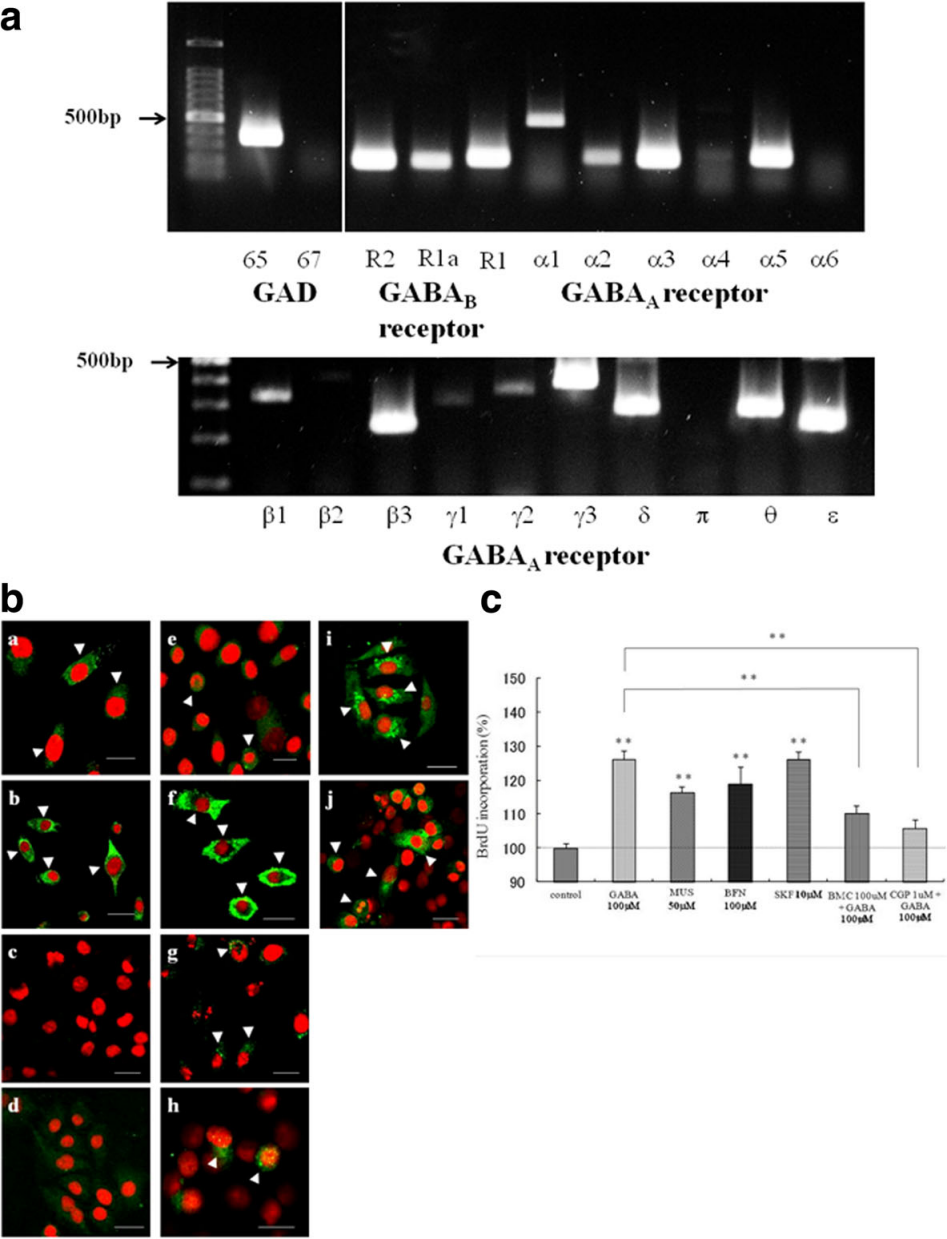
Expression of the GABAergic system and cell viability assay in OUMS-27 cells. **a** Determination of the mRNA levels of GAD65, GAD67, the GABA_A_ α1–6, β1–3, γ1–3, δ, π, θ, and ε subunits, and GABA_B_ R1a, R1a/b, and R2 in OUMS-27 cells by RT-PCR. **b** Confocal microscopy of the GABA, GAD, GABA_A_ receptor subunits, and GABA_B_ receptor subunits in OUMS-27 cells (**a- j**). (**a**) GABA, (**b**) GAD65, (**c**) GAD 67, (**d**) goat IgG, (**e**) α2, (**f**) α3, (**g**) β1, (**h**) γ3 (**i**) R1, and (**j**) R2. Immunoreactivity is visible as green fluorescence and cell nuclei are stained with PI (red). Arrow heads indicate immunoreactive cells. Scale bar = 10 μm. **c** Cell viability assay; OUMS-27 cells were treated with 100 μM GABA, 50 μM MUS (GABA_A_ receptor agonist), 100 μM BFN and 10 μM SKF (GABA_B_ receptor agonists), 100 μM GABA+ 100 μM BMC (GABA_A_ receptor antagonist) or 100 μM GABA+ 1 μM CGP (GABA_B_ receptor antagonist). The cell proliferation ELISA and BrdU assays were performed after drug treatment. Colorimetric analysis was performed using an ELISA plate reader. ** indicates significant differences between the control and each group (*P* < 0.01). Data are presented as the mean ± SD

We detected specific mRNA expression of GAD65, but not GAD67, in OUMS-27 cells. The mRNA expression of GABA_A_ receptor subunits α1, α2, α3, α5, β1, β3, γ1–3, δ, θ, and ε and the GABA_B_ receptor subunits R1 and R2, were also detected (Fig. 1a). In addition, immunohistochemistry revealed that GABA, GAD65, α2, α3, β1, and γ3 subunits of the GABA_A_ receptor, and the R1 and R2 subunits of the GABA_B_ receptor were expressed in the OUMS-27 cells (Fig. 1b).

### Incorporation of BrdU by chondrosarcoma cells treated with agonists and antagonists of GABA receptors

BrdU incorporation into OUMS-27 cells treated with 100 μM GABA, the GABA_A_ receptor agonist, 50 μM MUS and the GABA_B_ receptor agonists, 100 μM BFN and 10 μM SKF were significantly increased. However, the proliferation of the OUMS-27 cells treated with 100 μM GABA was significantly inhibited by the GABA_A_ receptor antagonist, 100 μM BMC and the GABA_B_ receptor antagonist, 1 μM CGP (Fig. 1c).

### Flow cytometric analysis quantitatively assessed apoptosis in CGP-treated chondrosarcoma cells

**Fig. 2:**
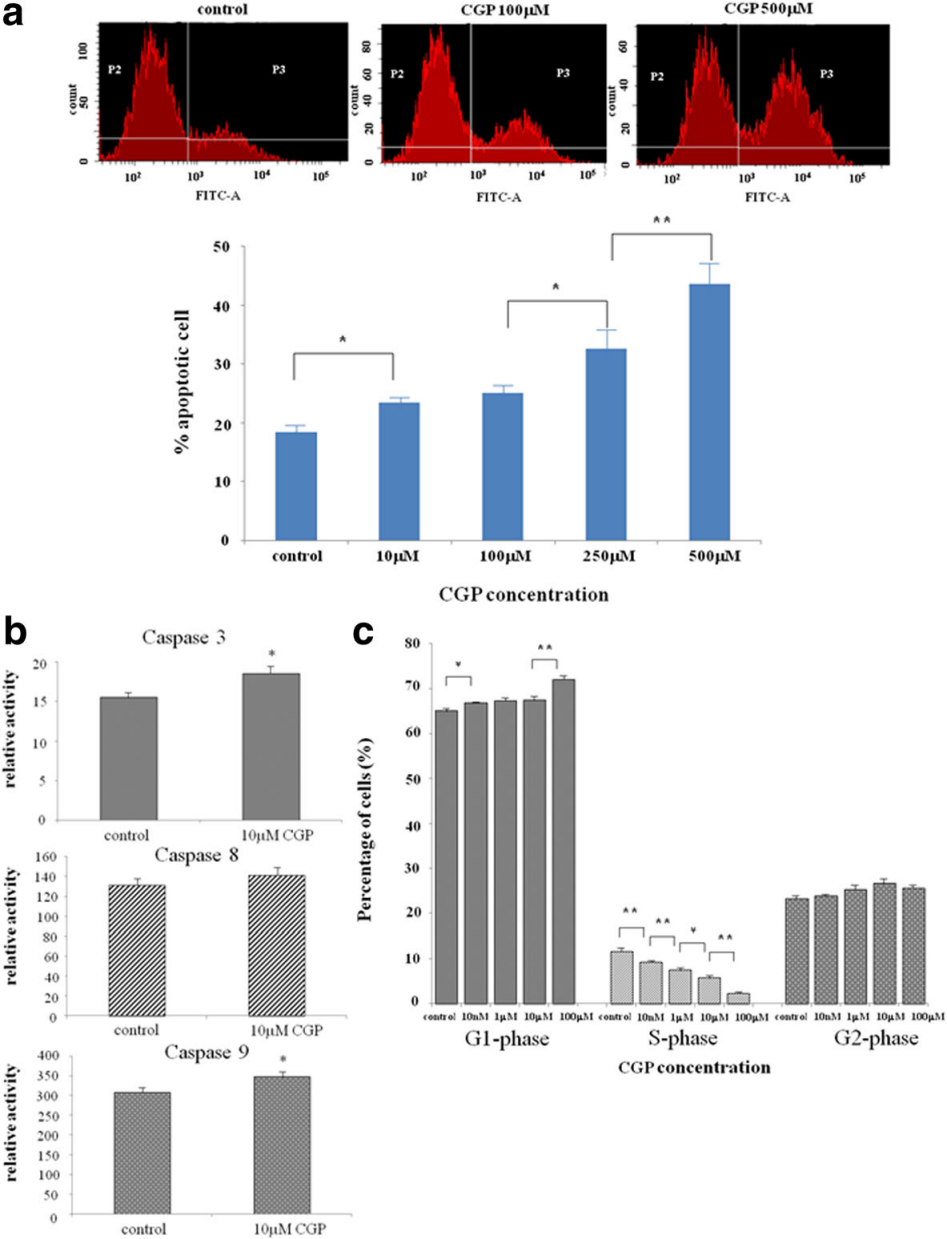
Apoptosis and cell cycle of OUMS-27 cells in vitro. **a** Flow cytometric analysis of apoptosis. OUMS-27 cells were treated with the indicated concentrations of CGP. Apoptotic cells were analyzed by FACScan flow cytometry. * indicates significant differences between the control and each group (*P* < 0.05). ** indicates significant differences between the control and each group (P < 0.01). **b.** Determination of caspase activity. OUMS-27 cells were treated with 10 μM CGP or DMSO. After 24 h of drug treatment, fluorescent intensities indicating cell viability and luminescent intensities indicating caspase-3, caspase-8, and caspase-9 activities were measured. Each estimate of caspase activity was adjusted with the corresponding cell viability. * indicates significant differences between the control and each group (P < 0.05). **c.** Determination of the percentage of cells in each phase of the cell cycle. OUMS-27 cells were treated with the indicated concentration of CGP for 24 h. The cell cycle distribution was measured by flow cytometry. * indicates significant differences between the control and each group (P < 0.05). ** indicates significant differences between the control and each group (P < 0.01). Data are presented as the mean ± SD

We performed flow cytometric analysis to quantitatively assess apoptosis in the OUMS-27 cells treated with CGP. The percentage of apoptotic (TUNEL- positive) cells significantly increased in response to CGP treatment in a dose-dependent manner (Fig. 2a).

### Activities of caspase 3, caspase 8, and caspase 9 in CGP-treated chondrosarcoma cells

To clarify the mechanism by which apoptosis was induced by CGP, we examined the activities of caspase 3, caspase 8, and caspase 9 in CGP-treated OUMS-27 cells using a cell viability assay and caspase luminescent assay. The activities of caspase 3 and caspase 9 were significantly elevated in cells treated with CGP for 24 h compared to in control cells. The activity of caspase 8 did not differ significantly between control and CGP-treated cells. However, caspase 8 tended to be up-regulated in CGP-treated cells (Fig. 2b). These results indicate that the GABA_B_ receptor antagonist inhibited cell proliferation via apoptotic pathways.

### Flow cytometric analysis of cell cycle in CGP-treated chondrosarcoma cells

We performed flow cytometric analysis to determine the percentage of OUMS-27 cells in each phase of the cell cycle after CGP treatment. The percentage of cells in G1 phase increased and those in S phase decreased significantly in a CGP dose-dependent manner (Fig. 2c). These results indicate that the GABA_B_ receptor antagonist induced G1 phase arrest and S phase suppression in the cell cycle.

### Western blot analysis of AKT/PI3K and mitogen-activated protein kinase (MAPK) levels in CGP-treated chondrosarcoma cells

**Fig. 3:**
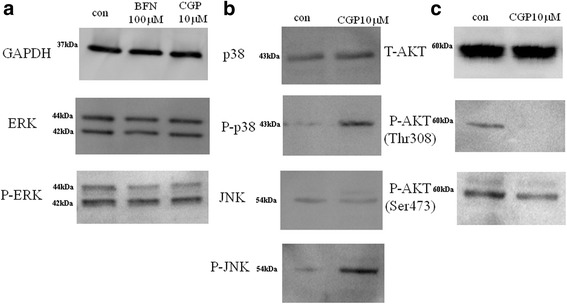
Western blot analysis for AKT/PI3K and MAPKs pathways. The levels of (**a**) GAPDH, ERK, and phospho-ERK in OUMS-27 cells treated with 100 μΜ BFN or 10 μΜ CGP (**b**) p38, phospho-p38, JNK, and phospho-JNK, and (**c**) total AKT, phospho-AKT (Th308) and phospho-AKT (Ser 473) treated with 10 μΜ CGP. The analysis was performed using respective primary antibodies and HRP-linked secondary antibodies. Protein levels were detected using a lumino image analyzer

The activation of MAPKs such as ERK, p38, and JNK, and PI3K/AKT/mTOR was examined by western blot analysis using antibodies that specifically recognize the phosphorylated forms of these proteins. The activities of ERK and phospho-ERK did not change after CGP or BFN treatment (Fig. 3a). However, MAPKs such as p38 and JNK were activated after CGP treatment (Fig. 3b). There were no apparent differences in total Akt levels between CGP-treated and control cells. The activities of both phospho-Akt-Thr308 and phospho-Akt-Ser473 in CGP-treated OUMS-27 cells were suppressed compared to in control cells (Fig. 3c).

### Real time PCR analysis of cell cycle-related genes

**Fig. 4:**
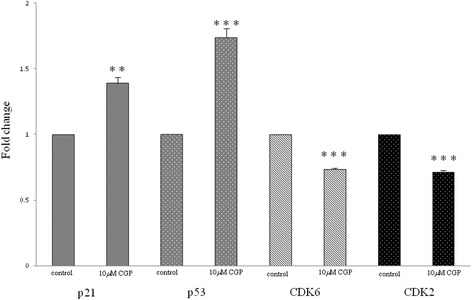
Real-time PCR analysis for cell cycle-related genes. The OUMS-27 cells were treated with 10 μM CGP for 24 h. Total RNA was extracted and cDNA was synthesized by reverse transcription. Primers for genes involved in cell cycle regulation were used and real-time PCR was performed. Data were corrected against GAPDH values. ** indicates significant differences between the control and each group (P < 0.01). *** indicates significant differences between the control and each group (*P* < 0.001). Data are presented as the mean ± SD

We investigated the mRNA levels of cell cycle-regulatory genes in OUMS-27 cells treated with CGP and control cells, as CGP treatment induced cell cycle arrest in the previous experiment. Real-time analysis revealed that the expression of p21 and p53 was significantly higher and that of cyclin–dependent kinase (CDK)6 and CDK2 was significantly lower in OUMS-27 cells treated with CGP compared to in control cells (Fig. 4).

### Ca^2+^ channel current recording

**Fig. 5:**
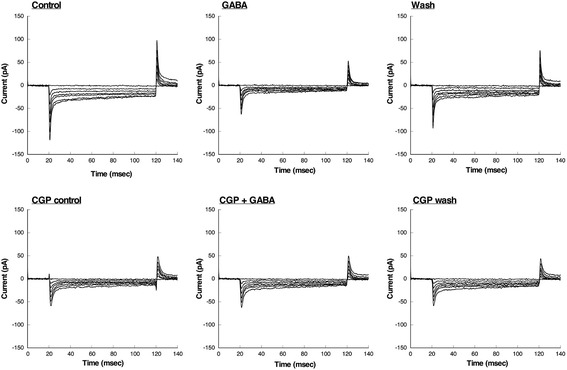
Whole-cell patch-clamp recordings for Ca^2+^ channel currents in OUMS-27 cells treated with 100 μM GABA and 1 μM CGP. The application of 100 μM GABA to the bath induced inhibition of Ca^2+^ currents. The Ca^2+^ currents were regained after GABA was washed away. Additionally, the inhibitory effect of GABA on Ca^2+^ current was attenuated by 1 μM CGP, which is GABA_B_ receptor antagonist

We performed whole-cell patch-clamp recordings of membrane Ca^2+^ channel currents in drug-treated OUMS-27 cells. The application of 100 μM GABA to the bath inhibited the Ca^2+^ currents. The Ca^2+^ currents were reproducibly regained after washing away the GABA. Additionally, the inhibitory effect of GABA on Ca^2+^ currents was attenuated by 1 μM CGP, which is a GABA_B_ receptor antagonist (Fig. 5).

### Intracellular Ca^2+^ measurement in chondrosarcoma cells

**Fig. 6:**
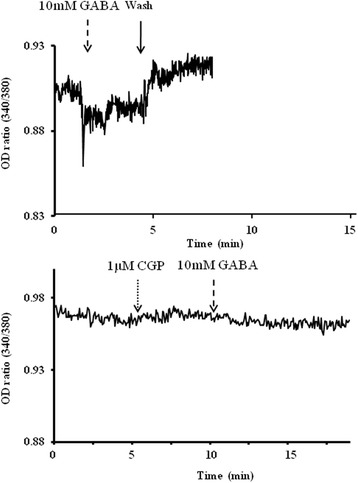
Intracellular Ca^2+^ measurement in chondrosarcoma cells. [Ca^2+^]_I_ in OUMS-27 cells treated with each drug was measured by Fura-2 AM, a calcium-sensitive dye. The relative transient calcium (OD_340nm_/OD_380nm_ excitation ratio) was recorded before and after the addition of 10 mM GABA. The recording was continued after GABA was washed out of the buffer. [Ca^2+^]_I_ in 1 μM CGP pretreated-OUMS-27 cells was recorded similarly after application of 10 mM GABA

[Ca^2+^]_I_ decreased immediately after inhibition of Ca^2+^ influx in response to 10 mM GABA in OUMS-27 cells. However, the [Ca^2+^]_I_ level increased with Ca^2+^ influx after washing. [Ca^2+^]_I_ did not change following application of 10 mM GABA after pretreatment with 1 μM CGP (Fig. 6).

## Discussion

Functional GABA_A_ receptors are composed of five types of subunits, and the subunit composition determines the properties of the receptors [7]. For example, functional GABA_B_ receptors require heterodimerization of the R1 and R2 subunits [29]. We first examined the expression of various components of the GABAergic system in the high-grade chondrosarcoma cell line OUMS-27. The RT-PCR results showed that OUMS-27 cells expressed GAD65 (Fig. 1a). GABA is synthesized from glutamate by GAD, which exists as two isoforms in mammals, with molecular weights of 65 kDa (GAD65) and 67 kDa (GAD67). GAD65 and GAD67 are encoded by two distinct genes located on human chromosomes 10 and 12, respectively [7]. We also detected the GABA_A_ receptor α1, α2, α3, α5, β1, β3, γ1–3, and δ, θ, ε subunits, as well as the GABA_B_ receptor R1 and R2 subunits (Fig. 1a). Immunohistochemical analyses revealed that GABA, GAD65, the α2, α3, β1 and γ 3 subunits of the GABA_A_ receptor, and the R1 and R2 subunits of the GABA_B_ receptor were expressed in OUMS-27 cells (Fig. 1b). Immunohistochemical analyses indicated that functionally active GABA_A_ and GABA_B_ receptors were present in OUMS-27 cells.

The effects of GABA_A_ receptor agonists [20, 30] and antagonists [31] reportedly vary in different types of cancer cells. Similarly, several reports indicated that GABA_B_ receptor agonists and GABA inhibited cell proliferation and migration in gastric cancer [32], colon cancer [33], and malignant hepatocytes [34, 35]. However, other reports showed that a GABA_B_ receptor antagonist inhibited cell proliferation and migration in breast cancer metastasis [36], renal cell carcinoma [37], and prostate cancer metastasis [31, 38]. Next, we examined the in-vitro effects of the GABAergic system in OUMS-27 cells. OUMS-27 cells treated with GABA, GABA_A_, and GABA_B_ receptor agonists showed significantly high BrdU incorporation. However, the proliferation of GABA-treated OUMS-27 cells was significantly inhibited by GABA_A_ and GABA_B_ receptor antagonists (Fig. 1c), demonstrating that GABA receptor antagonists inhibited the proliferation of OUMS-27 cells. These present data suggest that GABA_A_ and GABA_B_ receptors have physiologically distinct effects in different cancer cells. However, the mechanisms of these differential effects are unclear [36].

Here, we focused on the GABA_B_ receptor and its antagonists as GABA_B_ receptors participate in the signal transduction system [36] involving Ca^2+^ channels [20]. To investigate the underlying mechanisms by which tumor proliferation was inhibited by CGP, a GABA_B_ receptor antagonist, we performed a flow cytometric analysis of apoptosis. The percentage of apoptotic (TUNEL-positive) cells increased significantly after CGP treatment in a dose-dependent manner (Fig. 2a). To determine the mechanism by which CGP induced apoptosis, we analyzed caspase 3, caspase 8, and caspase 9 activities in OUMS-27 cells after CGP treatment in a cell viability assay and caspase luminescent assay. The activities of caspase 3 and 9 were significantly elevated in cells treated with CGP for 24 h compared to in control cells. However, caspase 8 tended to be up-regulated in CGP-treated cells, but the difference was not significant (Fig. 2b). These results suggest that both the mitochondria and death receptor pathways or mitochondria pathway alone are involved in CGP-induced apoptosis [39–41]. Cell cycle analyses showed that the percentage of cells in G1 phase increased, while that in S phase decreased significantly in CGP-treated cells in a dose-dependent manner (Fig. 2c). These results indicate that the GABA_B_ receptor antagonist inhibited cell proliferation via apoptotic pathways [42] and induced G1 phase arrest and S phase suppression [42–44]. An anti-proliferative effect via blocking of cell cycle progression has been observed in other cancer cells [39, 43, 44]. For example, treatment with a new bisphosphonate drug known as minodronate inhibited cell proliferation and induced S phase arrest in the chondrosarcoma cell line SW1353 [4, 40]. Furthermore, proline-rich polypeptide 1 cytokine of hypothalamic cytokines inhibited cell proliferation in the chondrosarcoma cell line JJ012 by suppressing cell cycle progression [45].

Signal transduction involving MAPK pathway components such as ERK, p38, and JNK and the PI3K/AKT/mTOR pathway contribute to the oncogenic process of the induction of apoptotic cell death and cell cycle entry [42, 43, 46]. In the present study, ERK and phospho-ERK activities were not changed in CGP-treated cells. However, p38 and JNK were activated. There were no apparent differences in total Akt levels in CGP-treated cells. The activities of both phospho-Akt-Thr308 and phospho-Akt-Ser473 in CGP-treated cells were suppressed compared to in controls (Fig. 3). The resistance of cells to targeted inhibition of either the MAPK or the PI3K/AKT/mTOR pathway can be partially explained as follows. Because the MAPK and PI3K/AKT/mTOR pathways are regulated by various feedback loops and cross-talk in the network, inhibition of one pathway results in activation of the other pathway via a compensatory mechanism. However, combination therapy using dual inhibitors of the MAPK and PI3K/AKT/mTOR pathways will be more effective for inhibiting proliferation and differentiation of tumor cells [47–49].

CGP-induced cell cycle arrest results from the activation of MAPK pathway members such as p38-and JNK [50, 51] and suppression of the PI3K/AKT/mTOR pathway. The MAPK and PI3K/AKT/mTOR pathways regulate the cell cycle by phosphorylating CDKs [50, 52–54]. Because CGP treatment induced cell cycle arrest, we investigated the mRNA levels of certain cell cycle-regulatory genes in CGP-treated cells. Real-time analysis revealed that the levels of CDKs such as CDK2 and CDK6 were reduced, while those of CDK inhibitors such as p21^Cip1^ and p53 were increased significantly in CGP-treated cells (Fig. 4). p53 is a tumor suppressor gene that is essential for inducing apoptosis and regulating cell cycle progression and DNA repair [46]. Many tumors exhibit mutations and deficiencies of p53 [55]. p53, which modulates the mitochondrial apoptotic pathway, plays important roles in regulating cell cycle progression, DNA repair, and cell death [42, 46, 56]. The importance of the p53 pathway in cell cycle progression in chondrosarcoma cells has been described in several reports [57, 58]. The high-grade chondrosarcoma cell line OUMS-27 with p-53 mutation was established by Kunisada et al. [21]. In the present study, in OUMS-27 cells with p-53 mutation, p-53 was activated by administration of CGP. Our results suggested that p53-related genes besides p53 gene were activated instead of mutated p53 [56, 59] or p53 wild-type allele affected p53 activation [56, 60]. However, the precise mechanism that p-53 was activated by administration of CGP was unclear [42]. Overall, these findings indicate that CGP administration induces cell cycle arrest in G1/G0 phase by increasing the levels of CDK inhibitors such as p21^Cip1^ and p53 [42, 43].

In the central nervous system, GABA_B_ receptors modulate Ca^2+^ channel currents via G-protein-coupled mechanisms. The Ca^2+^ channel currents were inhibited by baclofen, a GABA and GABA_B_ receptor agonist, and are involved changes in voltage dependence [61, 62]. In articular cartilage and chondrosarcoma, free Ca^2+^ or Ca^2+^ channels play important roles in the proliferation and differentiation of chondrocytes [63, 64].

Generally, there are various types of Ca^2+^-permeable channels such as transient receptor potential (TRP) channels, voltage-gated channels, and ligand-gated channels. In the present study, only GABA_B_ receptor operated-Ca^2+^ channels were closed by administration of GABA from compared to its normal state, influx of extracellular Ca^2+^ was stopped and intracellular Ca^2+^ concentration was expected to decrease (Fig. 5 and Fig. 6). Furthermore, addition of the GABA_B_ receptor antagonist maintained Ca^2+^ influx because inactivation of the GABA_B_ receptor ligand-operated channels did not occur (Fig. 6). The concentration of intracellular Ca^2+^ affects the influx of extracellular Ca^2+^ via GABA_B_ receptor-related Ca^2+^ channels in the plasma membrane and release of Ca^2+^ from intracellular stores such as the mitochondria and endoplasmic reticulum in response to stimulation of the cell death process [65–67]. However, we did not differentiate between Ca^2+^ channels related to the GABA_B_ receptors and other ligand-operated Ca^2+^ channels. The Ca^2+^ release mechanism from intracellular stores was unclear in the present study. Weak apoptosis occurred in glutamate-injured hippocampal neurons through inhibition of Ca^2+^ influx and caspase 3 activity after pyrroloquinoline quinone treatment [68]. Furthermore, the role of Ca^2+^ channels such as the TRPC Ca^2+^ channels and voltage-dependent Ca^2+^ channels in tumor cell growth in glioblastoma and prostate cancers has been described previously [50, 69–71]. In addition, Ca^2+^ channels regulate cartilage proliferation via influx of Ca^2+^ [63, 64]. Intracellular Ca^2+^ initiates various signaling pathways associated with cellular processes such as proliferation, differentiation, and apoptosis [70]. Changes in the concentration of intracellular Ca^2+^ activate cell proliferation, differentiation, and migration in certain tumor cells [50, 71]. Free intracellular Ca^2+^ regulates the MAPK and PI3K/AKT/mTOR pathways as a second messenger, and thereby affects cell death and the cell cycle in tumor cells [50]. Our results indicate that changes in intracellular Ca^2+^ levels via GABA_B_ receptor-related Ca^2+^ channels induces and modulates apoptotic signaling pathways in high-grade chondrosarcoma cells [70].

Chondrosarcoma originate from neural crest cells outside the mesoderm during embryogenesis [18, 19]. The GABAergic system is thought to play important roles in proliferation, migration, and differentiation during nervous system development. The response of the neurotrophic factors to GABA appears to act via the three GABA receptors. These findings suggest that the GABAergic system, which is an important nerve growth factor in neural crest development, may play important functional roles in the proliferation of chondrosarcoma. The GABAergic system can be utilized to develop new therapies for high-grade chondrosarcoma.

## Conclusions

Our study revealed that the GABA_B_ receptor antagonist had anti-tumor effects in OUMS-27 cells, a high-grade chondrosarcoma cell line, through cell cycle arrest at G1/S phase and induced apoptosis via dual inhibition of the PI3/Akt/mTOR and MAPK signaling pathways. In addition, changes in intracellular Ca^2+^ via the GABA_B_ receptor lead to inhibition of tumor proliferation in OUMS-27 cells by inducing and modulating apoptotic signaling pathways. The poor prognosis of patients with high-grade chondrosarcoma is expected to improve with this promising new therapy.

## Abbreviations

BrdU: 5-bromo-2′- deoxyuridine
CDK: cyclin-dependent kinase
DMSO: dimethyl sulfoxide
ELISA: enzyme-linked immunosorbent assay
GABA: gamma-aminobutyric acid
GAD: glutamic acid decarboxylase
MAPK: mitogen activated protein kinase
PBS: phosphate-buffered saline
RT: room temperature
SDS: sodium dodecyl sulfate
TUNEL: terminal deoxynucleotidyl transferase-mediated dUTP nick end-labeling

## References

[1] Hogendoorn PCW, Bovee JVMG, Nielsen GP, Bridge JA, Hogendoorn PCW (2013). Chondrosarcoma (grades I-III), including primary and secondary variants and periosteal chondrosarcoma. Fletvher CDM.

[2] de Jong Y, van Maldegem AM, Marino-Enriquez A, de Jong D, Suijker J, Briaire-de Bruijn IH, Kruisselbrink AB (2016). Inhibition of Bcl-2 family members sensitizes mesenchymal chondrosarcoma to conventional chemotherapy: report on a novel mesenchymal chondrosarcoma cell line. Lab Investig.

[3] Zhang YX, van Oosterwijk JG, Sicinska E, Moss S, Remillard SP, van Wezel T (2013). Functional profiling of receptor tyrosine kinases and downstream signaling in human chondrosarcomas identifies pathways for rational targeted therapy. Clin Cancer Res.

[4] Chow WA (2007). Update on chondrosarcomas. Curr Opin Oncol.

[5] Wolf P, Olpe HR, Avrith D, Haas HL (1978). GABAergic inhibition of neurons in the ventral tegmental area. Experientia.

[6] Schousboe A, Redburn DA (1995). Modulatory actions of gamma aminobutyric acid (GABA) on GABA type a receptor subunit expression and function. J Neurosci Res.

[7] Watanabe M, Maemura K, Kanbara K, Tamayama T, Hayasaki H. GABA and GABA receptors in the central nervous system and other organs. Int Rev Cytol. 2002;213:1–47. 10.1016/s0074-7696(02)13011-7 11837891

[8] Fiszman ML, Schousboe A (2004). Role of calcium and kinases on the neurotrophic effect induced by gamma-aminobutyric acid. J Neurosci Res.

[9] Magnaghi V, Ballabio M, Cavarretta IT, Froestl W, Lambert JJ, Zucchi I (2004). GABAB receptors in Schwann cells influence proliferation and myelin protein expression. Eur J Neurosci.

[10] Tamayama T, Maemura K, Kanbara K, Hayasaki H, Yabumoto Y, Yuasa M, et al. Expression of GABA(A) and GABA(B) receptors in rat growth plate chondrocytes: activation of the GABA receptors promotes proliferation of mouse chondrogenic ATDC5 cells. Mol Cell Biochem. 2005;273:117–26. 10.1007/s11010-005-8159-6 16013446

[11] Xilouri M, Papazafiri P (2006). Anti-apoptotic effects of allopregnanolone on P19 neurons. Eur J Neurosci.

[12] Katarova Z, Sekerková G, Prodan S, Mugnaini E, Szabó G (2000). Domain-restricted expression of two glutamic acid decarboxylase genes in midgestation mouse embryos. J Comp Neurol.

[13] Mackey HM, Payette RF, Gershon MD (1988). Tissue effects on the expression of serotonin, tyrosine hydroxylase and GABA in cultures of neurogenic cells from the neuraxis and branchial arches. Development.

[14] Alberti C (2010). Neuroendocrine differentiation in prostate carcinoma: focusing on its pathophysiologic mechanisms and pathological features. G Chir.

[15] Tamayama T, Kanbara K, Maemura K, Kuno M, Watanabe M (2001). Localization of GABA, GAD65 and GAD67 in rat epiphyseal growth plate chondrocytes. Acta Histochem Cytochem.

[16] Bowery NG, Enna SJ (2000). Gamma-aminobutyric acid(B) receptors: first of the functional metabotropic heterodimers. J Pharmacol Exp Ther.

[17] Calver AR, Robbins MJ, Cosio C, Rice SQ, Babbs AJ, Hirst WD (2001). The C-terminal domains of the GABA(b) receptor subunits mediate intracellular trafficking but are not required for receptor signaling. J Neurosci.

[18] Siar CH, Ha KO, Aung LO, Nakano K, Tsujigiwa H, Nagatsuka H (2010). Immunolocalization of notch signaling protein molecules in a maxillary chondrosarcoma and its recurrent tumor. Eur J Med Res.

[19] Cummings TJ, Shea CR, Reed JA, Burchette JL, Prieto VG (2000). Expression of the intermediate filament peripherin in extraskeletal myxoid chondrosarcoma. J Cutan Pathol.

[20] Watanabe M, Maemura K, Oki K, Shiraishi N, Shibayama Y, Katsu K (2006). Gamma-aminobutyric acid (GABA) and cell proliferation: focus on cancer cells. Histol Histopathol.

[21] Kunisada T, Miyazaki M, Mihara K, Gao C, Kawai A, Inoue H (1998). A new human chondrosarcoma cell line (OUMS-27) that maintains chondrocytic differentiation. Int J Cancer.

[22] Hayasaki H, Sohma Y, Kanbara K, Maemura K, Kubota T, Watanabe M. A local GABAergic system within rat trigeminal ganglion cells. Eur J Neurosci. 2006;23:745–57. 10.1111/j.1460-9568.2006.04602.x 16487155

[23] Tamura S, Watanabe M, Kanbara K, Yanagawa T, Watanabe K, Otsuki Y (2009). Expression and distribution of GABAergic system in rat knee joint synovial membrane. Histol Histopathol.

[24] Kanbara K, Mori Y, Kubota T, Watanabe M, Yanagawa Y, Otsuki Y (2011). Expression of the GABAA receptor/chloride channel in murine spermatogenic cells. Histol Histopathol.

[25] Shibata MA, Akao Y, Shibata E, Nozawa Y, Ito T, Mishima S (2007). Vaticanol C, a novel resveratrol tetramer, reduces lymph node and lung metastases of mouse mammary carcinoma carrying p53 mutation. Cancer Chemother Pharmacol.

[26] Hamill OP, Marty A, Neher E, Sakmann B, Sigworth FJ (1981). Improved patch-clamp techniques for high-resolution current recording from cells and cell-free membrane patches. Pflugers Arch.

[27] Delmas P, Abogadie FC, Dayrell M, Haley JE, Milligan G, Caulfield MP (1998). G-proteins and G-protein subunits mediating cholinergic inhibition of N-type calcium currents in sympathetic neurons. Eur J Neurosci.

[28] Wang F, Matsuoka N, Mutoh S, Kaneko S (2004). Modulation of Ca^2+^ channel currents by a novel antidementia drug N-(4-acetyl-1-piperazinyl)-p-fluorobenzamide monohydrate (FK960) in rat hippocampal neurons. J Pharmacol Exp Ther.

[29] White JH, Wise A, Main MJ, Green A, Fraser NJ, Disney GH (1998). Heterodimerization is required for the formation of a functional GABA(B) receptor. Nature.

[30] Zhang M, Gong Y, Assy N, Minuk GY (2000). Increased GABAergic activity inhibits alpha-fetoprotein mRNA expression and the proliferative activity of the HepG2 human hepatocellular carcinoma cell line. J Hepatol.

[31] Wu W, Yang Q, Fung KM, Humphreys MR, Brame LS, Cao A (2014). Linking γ-aminobutyric acid a receptor to epidermal growth factor receptor pathways activation in human prostate cancer. Mol Cell Endocrinol.

[32] Tatsuta M, Iishi H, Baba M, Nakaizumi A, Ichii M, Taniguchi H (1990). Inhibition by γ-amino-n-butyric acid and baclofen of gastric carcinogenesis induced by N-methyl-N’-nitro-N-nitrosoguanidine in wistar rats. Cancer Res.

[33] Tatsuta M, Iishi H, Baba M, Taniguchi H (1992). Attenuation by the GABA receptor agonist baclofen of experimental carcinogenesis in rat colon by azoxymethane. Oncology.

[34] Lodewyks C, Rodriguez J, Yan J, Lerner B, Lipschitz J, Nfon C (2011). GABA-B receptor activation inhibits the in vitro migration of malignant hepatocytes. Can J Physiol Pharmacol.

[35] Ortega A. A new role for GABA: inhibition of tumor cell migration. Trends Pharmacol Sci. 2003;24:151–4. 10.1016/S0165-6147(03)00052-X 12706998

[36] Zhang D, Li X, Yao Z, Wei C, Ning N, Li J (2014). GABAergic signaling facilitates breast cancer metastasis by promoting ERK1/2-dependent phosphorylation. Cancer Lett.

[37] Inamoto T, Azuma H, Sakamoto T, Kiyama S, Ubai T, Kotake Y (2007). Invasive ability of human renal cell carcinoma cell line Caki-2 is accelerated by gamma-aminobutyric acid, via sustained activation of ERK1/2 inducible matrix metalloproteinases. Cancer Investig.

[38] Azuma H, Inanoto T, Sakamoto T, Kiyama S, Ubai T, Shinohara Y (2003). γ-aminobutyric acid as a promoting factor of cancer metastasis; induction of matrix metalloproteinase production is potentially its underlying mechanism. Cancer Res.

[39] Kurose H, Shibata MA, Iinuma M, Otsuki Y (2012). Alterations in cell cycle and induction of apoptotic cell death in breast cancer cells treated with α-mangostin extracted from mangosteen pericarp. J Biomed Biotechnol 2012.

[40] Kubo T, Shimose S, Matsuo T, Tanaka K, Yasunaga Y, Sakai A (2006). Inhibitory effects of a new bisphosphonate, minodronate, on proliferation and invasion of a variety of malignant bone tumor cells. J Orthop Res.

[41] Jiao P, Zhou YS, Yang JX, Zhao YL, Liu QQ, Yuan C (2013). MK-2206 induces cell cycle arrest and apoptosis in HepG2 cells and sensitizes TRAIL-mediated cell death. Mol Cell Biochem.

[42] Cordero-Herrera I, Martín MA, Bravo L, Goya L, Ramos S (2013). Epicatechin gallate induces cell death via p53 activation and stimulation of p38 and JNK in human colon cancer SW480 cells. Nutr Cancer.

[43] Zhong D (2014). Gu C, Shi L, Xun T, li X, Liu S, et al. Obatoclax induces G1/G0-phase arrest via p38/p21^waf1/Cip1^ signaling pathway in human esophageal cancer cells. J Cell Biochem.

[44] Shibata MA, Ito Y, Morimoto J, Otsuki Y (2004). Lovastatin inhibits tumor growth and lung metastasis in mouse mammary carcinoma model: a p-53-independent mitochondrial-mediated apoptotic mechanism. Carcinogenesis.

[45] Galoian K, Temple TH, Galoyan A (2011). Cytostatic effect of the hypothalamic cytokine PRP-1 is mediated by mTOR and cMyc inhibition in high-grade chondrosarcoma. Neurochem Res.

[46] Shibata MA, Iinuma M, Morimoto J, Kurose H, Akamatsu K (2011). Okuno Y, et al. α-Mangostin extracted from the pericarp of the mangosteen (*Garcinia mangostana* Linn) reduces tumor growth and lymph node metastasis in an immunocompetent xenograft model of metastatic mammary cancer carrying a p53 mutation. BMC Med.

[47] Engelman JA, Chen L, Tan X, Crosby K, Guimaraes AR, Upadhyay R (2008). Effective use of PI3K and MEK inhibitors to treat mutant Kras G12D and PIK3CA H1047R murine lung cancers. Nat Med.

[48] Watson AL, Anderson LK, Greeley AD, Keng VW, Rahrmann EP, Halfond AL (2014). Co-targeting the MAPK and PI3K/AKT/mTOR pathways in two genetically engineered mouse models of schwann cell tumors reduces tumor grade and multiplicity. Oncotarget.

[49] Guenther MK, Graab U, Fulda S (2013). Synthetic lethal interaction between PI3K/Akt/mTOR and Ras/MEK/ERK pathway inhibition in rhabdomyosarcoma. Cancer Lett.

[50] Song M, Chen D, Yu SP. The TRPC channel blocker SKF 96365 inhibits glioblastoma cell growth by enhancing reverse mode of the Na^+^/Ca^2+^ exchanger and increasing intracellular Ca^2+^. Br J Pharmacol. 2014;171:3432–47. 10.1111/bph.12691 PMC4105931 24641279

[51] Miyake T, Shirakawa H, Kusano A, Sakimoto S, Konno M, Nakagawa T (2014). TRPM2 contributes to LPS/IFNγ-induced production of nitric oxide via the p38/JNK pathway in microglia. Biochem Biophys Res Commun.

[52] Lin YC, Liang YC, Sheu MT, Lin YC, Hsieh MS, Chen TF (2008). Chondroprotective effects of glucosamine involving the p38 MAPK and Akt signaling pathways. Rheumatol Int.

[53] Lu X, Tang X, Guo W, Ren T, Zhao H (2010). Sorafenib induces growth inhibition and apoptosis of human chondrosarcoma cells by blocking the RAF/ERK/MEK pathway. J Surg Oncol.

[54] Yang P, Wang G, Huo H, Li Q, Zhao Y, Liu Y (2015). SDF-1/CXCR4 signaling up-regulates survivin to regulate human sacral chondrosarcoma cell cycle and epithelial-mesenchymal transition via ERK and PI3K/AKT pathway. Med Oncol.

[55] O'Connor PM, Jackman J, Bae I (1997). Characterization of the p53 tumor suppressor pathway in cell lines of the National Cancer Institute anticancer drug screen and correlations with the growth-inhibitory potency of 123 anticancer agents. Cancer Res.

[56] Bertheau P, Plassa F, Espié M, Turpin E, de Roquancourt A, Marty M (2002). Effect of mutated TP53 on response of advanced breast cancers to high-dose chemotherapy. Lancet.

[57] Bovée JV, Hogendoorn PC, Wunder JS, Alman BA (2010). Cartilage tumours and bone development: molecular pathology and possible therapeutic targets. Nat Rev Cancer.

[58] Kumari R, Li H, Haudenschild DR, Fierro F, Carlson CS, Overn P (2012). The oncogene LRF is a survival factor in chondrosarcoma and contributes to tumor malignancy and drug resistance. Carcinogenesis.

[59] Vayssade M, Haddada H, Faridoni-Laurens L, Tourpin S, Valent A, Bénard J (2005). p73 functionally replaces p53 in Adriamycin-treated, p53-deficient breast cancer cells. Int J Cancer.

[60] Jackson JG, Pant V, Li Q, Chang LL, Quintás-Cardama A, Garza D (2012). p53-mediated senescence impairs the apoptotic response to chemotherapy and clinical outcome in breast cancer. Cancer Cell.

[61] Mintz IM, Bean BP (1993). GABA_B_ receptor inhibition of P-type Ca^2+^ channels in central neurons. Neuron.

[62] Filippov AK, Couve A, Pangalos MN, Walsh FS, Brown DA, Moss SJ (2000). Heteromeric assembly of GABA_B_R1 and GABA_B_R2 receptor subunits inhibits Ca^2+^ current in sympathetic neurons. J Neurosci.

[63] Funabashi K, Ohya S, Yamamura H, Hatano N, Muraki K, Giles W (2010). Accelerated Ca^2+^ entry by membrane hyperpolarization due to Ca^2+^-activated K^+^ channel activation in response to histamine in chondrocytes. Am J Physiol Cell Physiol.

[64] Cheng Z, Tu C, Rodriguez L, Chen TH, Dvorak MM, Margeta M, et al. Type B gamma-aminobutyric acid receptors modulate the function of the extracellular Ca2^+^-sensing receptor and cell differentiation in murine growth plate chondrocytes. Endocrinology. 2007;148:4984–92. 10.1210/en.2007-0653 17615148

[65] Maier PJ, Zemoura K, Acuña MA, Yévenes GE, Zeilhofer HU, Benke D (2014). Ischemia-like oxygen and glucose deprivation mediates down-regulation of cell surface γ-aminobutyric acid_B_ receptors via the endoplasmic reticulum (ER) stress-induced transcription factor CCAAT/enhancer-binding protein (C/EBP)-homologous protein (CHOP). J Biol Chem.

[66] Sato T, Kaneko YK, Sawatani T, Noguchi A, Ishikawa T (2015). Obligatory role of early Ca^2+^ responses in H_2_O_2_-induced β-cell apoptosis. Biol Pharm Bull.

[67] Chen L, Sun Q, Zhou D, Song W, Yang Q, Ju B (2017). HINT2 triggers mitochondrial Ca^2+^ influx by regulating the mitochondrial Ca^2+^ uniporter (MCU) complex and enhances gemcitabine apoptotic effect in pancreatic cancer. Cancer Lett.

[68] Zhang Q, Shen M, Ding M, Shen D, Ding F (2011). The neuroprotective action of pyrroloquinoline quinone against glutamate-induced apoptosis in hippocampal neurons is mediated through the activation of PI3K/Akt pathway. Toxicol Appl Pharmacol.

[69] Prevarskaya N, Skryma R, Bidaux G, Flourakis M, Shuba Y (2007). Ion channels in death and differentiation of prostate cancer cells. Cell Death Differ.

[70] Sergeev IN (2005). Calcium signaling in cancer and vitamin D. J Steroid Biochem Mol Biol.

[71] Zhang Y, Wang H, Qian Z, Feng B, Zhao X, Jiang X (2014). Low-voltage-activated T-type Ca^2+^ channel inhibitors as new tools in the treatment of glioblastoma: the role of endostatin. Pfluquers Arch.

